# Solitary retinal capillary hemangioma with combined retinal detachment- rare presentation

**Published:** 2019

**Authors:** S Hemant Trehan, Ashok Kumar, Vikas Ambiya, Gaurav Kapoor, K Raji, Amit Arora

**Affiliations:** * Army Hospital, Research & Referral, Delhi Cantt-10; ** Army College of Medical Sciences & Base Hospital, Delhi Cantt-10

**Keywords:** retinal capillary hemangioma, combined retinal detachment, endoresection, pars plana vitrectomy

## Abstract

**Objective.** To report a rare presentation of solitary retinal capillary hemangioma manifesting with combined retinal detachment as initial presentation and its successful management.

**Methods.** A 35-year-old healthy Indian male presented with combined retinal detachment associated with solitary retinal capillary hemangioma as initial presentation; a clinical entity still not reported in literature. Patient was managed with pars plana vitrectomy combined with retinectomy, endolaser, & silicon oil tamponade with good visual & anatomical recovery.

**Results.** Patient had good clinical outcome with final best-corrected visual acuity (BCVA) of 6/ 24 and well attached retina at last follow-up.

**Conclusion.** Solitary retinal capillary hemangiomas can rarely present with advanced vitreo-retinal complications like combined retinal detachment as initial manifestation that can be effectively managed with skilled & appropriate surgical intervention.

## Introduction

Retinal capillary hemangiomas are benign vascular tumors of the retina either occurring as isolated tumors or in association with tumors in other systems, especially central nervous system hemangioblastomas and renal cell carcinomas as part of von Hippel-Lindau (VHL) disease [**1**-**3**]. Retinal hemangiomas are mainly asymptomatic but can present with vision loss that occurs due to exudation at the macular region or secondary tractional retinal detachment, which occurs due to development of gliotic tissue involving the macula [**4**]. Different treatment modalities undertaken for treatment of retinal hemangiomas include laser photocoagulation, cryotherapy, photodynamic therapy (PDT), anti-VEGF, and vitreo-retinal surgery depending on clinical presentation with variable outcome in these patients [**5**-**9**]. 

We report a case of solitary retinal hemangioma that manifested with combined retinal detachment, a rare presenting feature of retinal hemangioma, and its successful management. To the best of our knowledge, this rare presentation has not been reported in published literature until now.

## Case Report

**Fig. 1:**
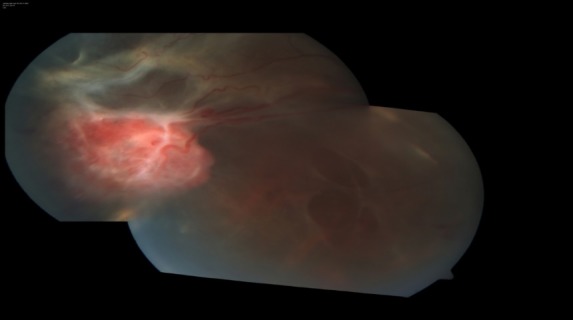
Fundus Picture of right eye showing total retinal detachment with about 6 Disc Diameter (DD) size reddish orange mass (blue arrow), with two large stretch breaks (yellow arrow) in inferotemporal quadrant and with surrounding fibrous tractions

**Fig. 2:**
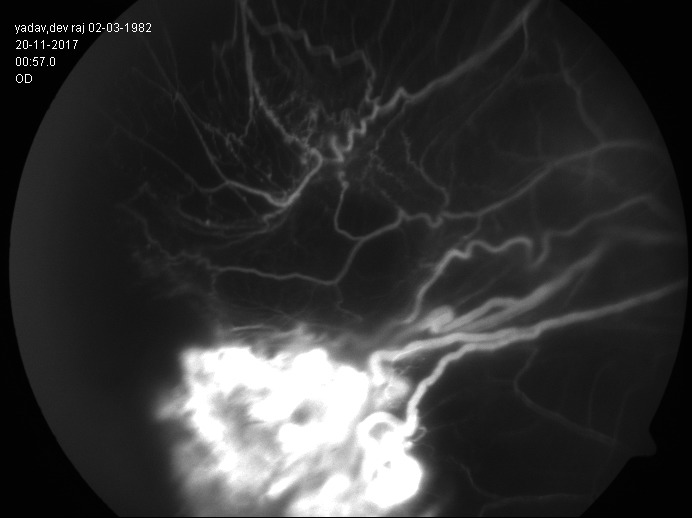
Fundus fluorescein angiography of the right eye showing typical feeder vessel (yellow arrow) with early hyperfluorescence of vascular mass (blue arrow) and draining vein

A 35-year-old, serving military soldier presented with diminution of vision in his right eye for the past 3 months, which has rapidly worsened over the last month. His systemic parameters were within normal limits. Ocular examination revealed a best-corrected visual acuity (BCVA) of hand movement close to face (HMCF) with accurate projection of rays in the right eye and 6/ 6 in the left eye. Examination of the anterior segment was normal in both eyes. Fundus examination in the right eye revealed total retinal detachment with about 6 Disc Diameter (DD) size reddish orange mass with two large stretch breaks stretching in the inferotemporal quadrant with surrounding fibrous tractions (**Fig. 1**) and examination of the left eye fundus was normal. We initially considered the differential diagnosis of retinal hemangioma and vasoproliferative tumors of the retina (VPRT). Fundus fluorescein angiography confirmed the diagnosis of retinal capillary hemangioma, showing typical feeder vessel with early hyperfluorescence of vascular mass and draining vein (**Fig. 2**) in the right eye, with no evidence of hemangioma in the left eye.

**Fig. 3:**
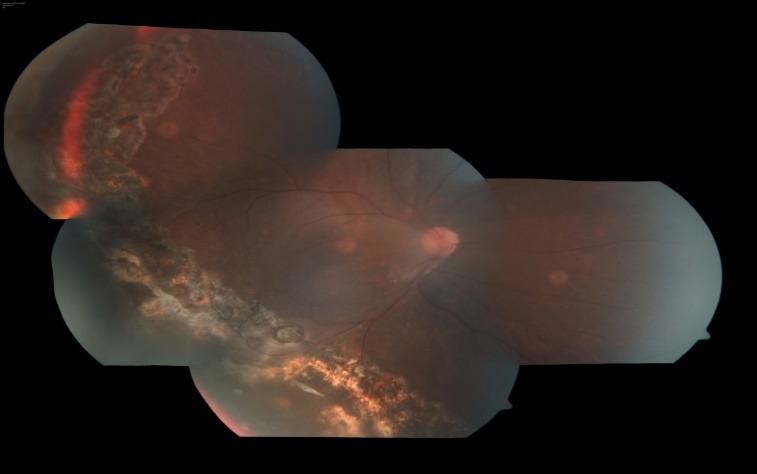
Fundus picture of the same patient 9 months post surgery, showing attached retina, no evidence of remnant of tumor/reproliferation and healthy well-lasered retinectomy site in temporal periphery

Since the patient had already reached a stage in which the treatment modalities like laser photocoagulation, cryotherapy, photodynamic therapy (PDT), anti-VEGF, did not have any role due to total retinal detachment, he underwent combined surgery with sclera band buckle (240 size) with pars plana vitrectomy and endoresection of tumor with high viscosity silicon oil tamponade. There were no intra-operative complications with stable course post surgery and attached retina. Silicon oil was removed 5 months after the initial surgery, as soon as the emulsification was noted. At 9 months post surgery, BCVA in right eye had improved to 6/ 24 with attached retina, no evidence of remnant of tumor/ reproliferation and healthy well laser retinectomy site in temporal periphery (**Fig. 3**), thus showing a successful outcome in unusual presentation. 

## Discussion

Retinal hemangiomas are benign vascular tumors that can rarely enlarge and produce visual loss by complicated retinal exudates, exudative retinal detachment, an epiretinal membrane, and vitreous hemorrhage [**10**,**11**]. Gaudric et al. described 23 eyes of 21 patients with retinal hemangioma with advanced complications like exudative or tractional retinal detachment managed with surgical intervention, but none of them had combined retinal detachment [**12**]. Kuo et al. described 13 eyes of 12 patients with retinal hemangioma, out of which 10 were located in the peripheral retina and none presented with combined retinal detachment [**13**]. 

Our patient presented with combined retinal detachment associated with retinal hemangioma, which was confirmed with typical features fluorescein angiography features like early hyperfluorescence of tumor with well-defined feeder and draining vessel. Retinal hemangioma was large, located at equator, with surrounding fibrous traction making surgical treatment difficult. Gaudric et al. treated 9 of 23 eyes with advanced complications with endoresection of tumor, which improved or stabilized visual functions in these patients but had a high incidence of recurrence/ reproliferation of tumor [**12**]. Mcdonald et al. described vitrectomy in 10 eyes with retinal hemangioma and macular pucker/ tractional retinal detachment with multiple complications like recurrent retinal detachment, non-regressed retinal angioma or epiretinal membrane formation [**14**]. However, none of these eyes had combined/ rhegmatogenous retinal detachments as initial or late presentation. 

Our patient underwent surgical intervention in the form of scleral band buckle (240 type) with pars plana vitrectomy, endoresection of tumor with high viscosity silicon oil tamponade. Patient had stable post-operative period with good visual recovery. He underwent silicon oil removal 5 months post initial surgery with good anatomical and visual outcome and no evidence of tumor proliferation/ redetachment at last follow-up, which was 9 months post vitrectomy.

Thus, combined retinal detachment can be an unusual or atypical presentation of rare etiology like retinal capillary hemangiomas but appropriate and skilled vitreo-retinal surgical intervention can lead to successful visual and anatomical outcomes in these patients. 

**Availability of Data & material**

Freely available on request.

**Competing Interest**

Nil.

**Conflict of Interest**

No conflict of interest was declared by the authors.

**Financial Disclosure**

The authors declared that this study received no financial support.

**Acknowledgement**

Nil.
